# A prospective single-center protocol for using near-infrared fluorescence imaging with indocyanine green during staging laparoscopy to detect small metastasis from pancreatic cancer

**DOI:** 10.1186/s12893-019-0635-0

**Published:** 2019-11-07

**Authors:** Sachiyo Shirakawa, Hirochika Toyama, Masahiro Kido, Takumi Fukumoto

**Affiliations:** Division of Hepato-Biliary-Pancreatic Surgery, Department of Surgery, Kobe University Graduate School of Medicine, 7-5-2 Kusunoki-cho Chuo-ku, Kobe Hyogo, Japan

**Keywords:** Pancreatic cancer, Indocyanine green, Liver metastasis, Peritoneal metastasis, Staging laparoscopy

## Abstract

**Background:**

Pancreatic resection and radiotherapy are powerful tools in the multidisciplinary local treatment of pancreatic ductal adenocarcinoma (PDAC). However, 10–20% of patients with preoperatively resectable PDAC have radiographically occult metastases, which results in laparotomy without resection. This study aims to explore the utility of intraoperative near-infrared (NIR) imaging with indocyanine green (ICG) during staging laparoscopy to detect PDAC metastasis.

**Methods:**

This prospective study will evaluate patients with radiographically non-metastatic PDAC before they undergo planned pancreatic resection or chemoradiotherapy. Enrolled patients will receive ICG intravenously (0.5 mg/kg) before the staging laparoscopy. During the staging laparoscopy, the abdominal cavity will be observed using standard white-light laparoscopic imaging and then using NIR-ICG imaging. Suspicious lesions that are detected using standard imaging and/or NIR-ICG imaging will be examined intraoperatively using frozen sections and permanent specimens. We will evaluate the benefit of NIR-ICG imaging based on its ability to identify additional liver or peritoneal lesions that were not detected during standard white-light imaging.

**Discussion:**

This study will help establish the clinical utility of NIR-ICG imaging to more precisely identify metastases from radiographically non-metastatic PDAC. This approach may help avoid needless major surgery or radiotherapy.

**Trial registration:**

This protocol was registered on April 1, 2017 on the UMIN Clinical Trials Registry: UMIN000025900 and February 26, 2019 on the Japan Registry of Clinical Trials: jRCT1051180076.

## Background

Pancreatic ductal adenocarcinoma (PDAC) is the fourth leading cause of cancer-related death in Japan, and increasing incidences have been reported in Japan and Western countries. Pancreatic resection and radiotherapy are powerful tools in the multidisciplinary local treatment of PDAC. However, in patients with preoperatively resectable PDAC (R-PDAC), radiographically occult metastases are noted in 10–20% of cases, with the metastatic lesions typically being detected in the liver or peritoneum. Staging laparoscopy is thought to be helpful for identifying small metastases and for reducing the rate of non-therapeutic laparotomy [1]. Staging laparoscopy has also revealed metastatic lesions in approximately 20% of patients with radiographically locally advanced unresectable PDAC (LA-PDAC) [2].

Despite an extensive work-up with staging laparoscopy, some patients with localized PDAC also experience early progression with distant metastasis. Local treatments (e.g., resection or radiotherapy) may not be indicated in these cases, and a more accurate detection of radiographically occult metastases may help improve treatment selection for these PDAC patients. Intraoperative near-infrared (NIR) visualization using indocyanine green (ICG) reportedly improves the diagnostic accuracy for radiographically occult tumors of the liver or peritoneum in cases with hepatocellular carcinoma or colorectal cancer [3–5]. However, there is a paucity of information regarding the role of NIR-ICG imaging in the work-up for PDAC. Therefore, the protocol for this prospective study was designed to explore the clinical utility of NIR-ICG imaging during staging laparoscopy to detect PDAC metastasis.

## Methods

### Study design

This single-center prospective study will evaluate patients with radiographically R-PFAC or LA-PDAC before they undergo planned local treatment (pancreatic resection or chemoradiotherapy). Enrolled patients will receive ICG preoperatively and undergo staging laparoscopy with standard white-light imaging followed by NIR-ICG imaging at the Kobe University Hospital. The study protocol has been approved by the Ethics Committee at Kobe University Medical School Hospital (approval No: 290078) and the Kobe University Clinical Research Ethical Committee (approval No: C180070), and the study was opened in March 2018.

### Primary outcome

The primary outcome will be the ability of NIR-ICG imaging: the efficacy is defined as an identification at least one metastatic tumor that was not detected using standard white-light imaging. This outcome was selected based on a desire to obtain additional information that is not captured during standard imaging.

### Secondary outcome

The secondary outcome will be any change in treatment that was based on the findings from the NIR-ICG imaging.

### Study population

Planned local treatment (pancreatic resection or radiotherapy) for radiographically T3 or T4 (UICC) [6] tumors and written informed consent for staging laparoscopy are indications for staging laparoscopy.

#### Inclusion criteria

Patients will be included in the study if they fulfill the following criteria:

- Age of ≥20 years and able to provide written informed consent
- Pathological or clinical diagnosis of PDAC
- Radiographically non-metastatic disease
- Planned for staging laparoscopy followed by a local treatment (pancreatic resection or radiotherapy).

#### Exclusion criteria

Patients will be excluded from the study if they fulfill any of the following criteria:

- Allergic to iodine
- Pregnancy
- Chronic liver disease with the Child-Pugh class B or C
- Radiographically suspected malignant ascites
- Another malignancy within five years other than endoscopically cured digestive tract cancer
- Investigator judgement that the patient should not participate for any reason

### Sample size

The target sample size is set to 40 patients in order to detect a significant difference in the primary outcome. Studies of NIR-ICG imaging for colorectal cancer have indicated that 10–43% of patients had additional metastases that were only identified using NIR-ICG imaging [7]. Thus, we conservatively assumed that NIR-ICG imaging would detect additional radiographically occult metastases in 10% of the cases. Thus, 7 patients would be needed to identify a significant difference in the primary outcome, based on a null hypothesis of 0% efficacy with a one-sided significance level of 2.5 and 80% power. Between April 2014 and June 2016, we retrospectively determined that the expected rate of radiographically occult PDAC metastases was approximately 20% at our center (26% of 72 historically study-eligible patients). Therefore, we calculated the required sample size to be 35 patients (7 / 0.2 = 35), and increased the sample size to 40 patients to account for 5 possible dropouts. Kobe University Hospital typically treats 20–30 patients per year who would be considered eligible for the study, which suggest that a sufficient number of patients may be enrolled during the recruitment period.

### Staging laparoscopy (intervention)

Enrolled patients will receive ICG intravenously (0.5 mg/kg) on the day before staging laparoscopy. During staging laparoscopy, peritoneal washing cytology (PWC) sample from the pelvis with 100 ml of normal saline is collected at first. The abdominal cavity will be observed using standard white-light laparoscopic imaging and then using NIR-ICG imaging. Suspicious lesions that are defined as detectable nodules under white-light imaging or NIR-ICG imaging on the liver or peritoneum, will be examined intraoperatively using frozen sections and permanent specimens. If more than five suspicious lesions are observed on the individual organs (liver and peritoneum), we will examine five lesions using frozen sections for each organ, and additional specimens of the frozen sections will be taken when all specimens are diagnosed as benign lesions in the first examination. We will resect up to ten lesions for each organ.

The findings will be evaluated intraoperatively by the entire operative team (two or three surgeons) and at least one other surgeon will postoperatively review the recorded video. We have defined the benefit of NIR-ICG imaging as the identification of additional liver or peritoneal lesions that were not identified during the standard white-light imaging. The final judgement will be based on consensus among the operative surgeons.

Each lesion that was taken during staging laparoscopy is examined using standard white-light imaging, NIR-ICG imaging, and histological finding to detect for positive or negative lesions. False positive or negative detection with white-light imaging and NIR-ICG imaging is recorded according to the confirmed findings of staging laparoscopy and histological diagnosis.

The staging laparoscopy will be performed using a laparoscopic high-definition fluorescence imaging system (Karl Storz GmbH & Co. KG, Tuttlingen, Germany). The system includes light sources for white-light and 760-nm light, as well as a 30° 10-mm laparoscope containing optical filters. The system allows the surgeon to easily switch between the white-light and NIR sources using a foot pedal.

### Therapeutic decision making

**Fig. 1:**
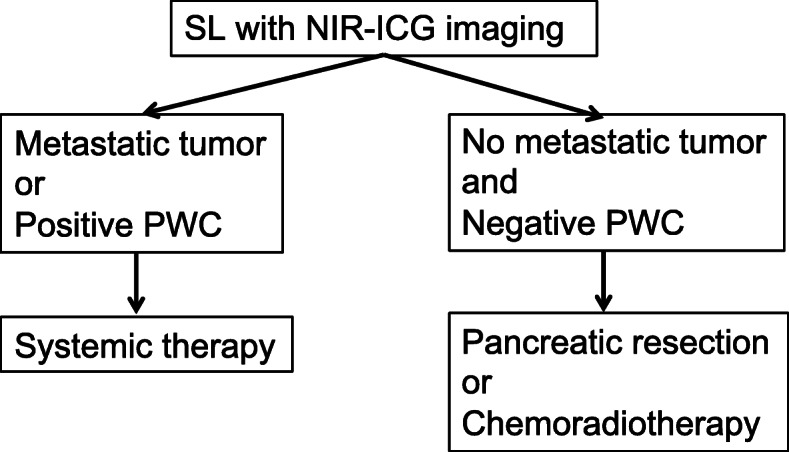
Therapeutic decision making by staging laparoscopy. SL: staging laparoscopy; PWC: peritoneal washing cytology

In this study, “metastatic tumor” is defined as histologically diagnosed lesions in permanent specimens regardless of the positive or negative detection using NIR-ICG imaging. Local treatment (surgery or radiotherapy) is cancelled for patients with metastatic tumor or positive PWC who are diagnosed using staging laparoscopy (Fig. 1).

### Schedule

**Fig. 2:**
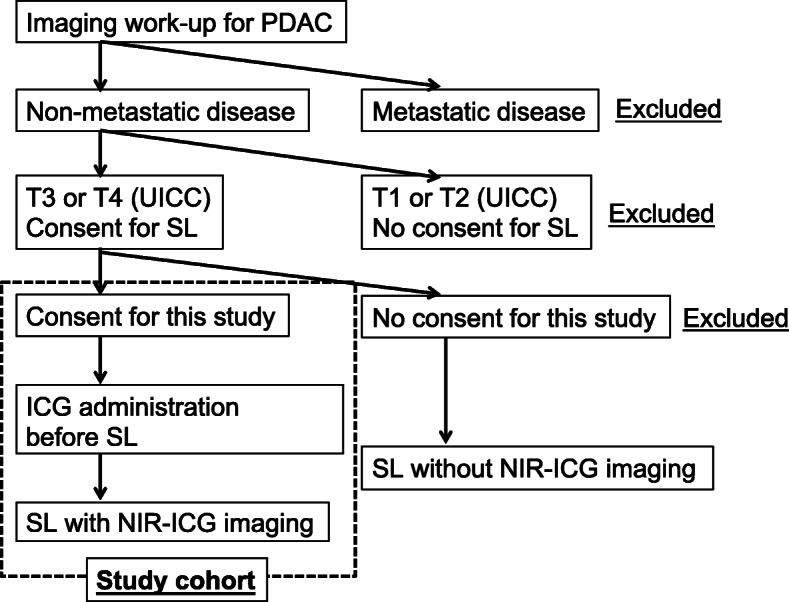
Flowchart for this study. PDAC: pancreatic ductal adenocarcinoma; SL: staging laparoscopy; PWC: peritoneal washing cytology

The flowchart of this study is shown in Fig. 2. Table 1 shows the schedule for the observations, tests, and assessments, which will be performed by the principal investigator or sub-investigators. There are no relevant restrictions regarding concomitant care or interventions that are prohibited during the trial. Patients may withdraw or be withdrawn from the study at any time, at which point the date of discontinuation, reason for discontinuation, clinical course, and safety and efficacy outcomes will be recorded in the patient’s medical record and case report form (CRF). The withdrawal criteria are:

- The patient wishes to withdraw from the study or withdraws their informed consent.
- The patient is found to be ineligible after their registration.
- Exacerbation of complications (e.g., previous disease) precludes further testing or evaluation.
- The patient is found to be pregnant.
- The study is terminated.

**Table 1:** Study steps and evaluations

	Study period			
	Enrolment	Intervention (staging laparoscopy)	Post-intervention	
			7 days	12 months
Informed consent	○			
Physical examination	○	○	○	○
Demographics and medical history	○			
Blood testing	○	○	○	○
Abdominal CECT	○			○
EOB-MRI	○			
FDG-PET	○			
Administration of ICG		○		
Adverse events		○	○	○
PDAC recurrence			○	○

*CECT* contrast-enhanced computed tomography, *EOB-MRI* gadolinium ethoxybenzyl diethylenetriamine pentaacetic acid-enhanced magnetic resonance imaging, *FDG-PET* 18F-flurodeoxyglucose positron emission tomography, *ICG* indocyanine green, *PDAC* pancreatic ductal adenocarcinoma

### Data collection

The principal investigator or sub-investigators will complete the CRFs, and the data manager will collect the CRFs to construct datasets for the statistical analysis. Safety information will be recorded as a secondary endpoint on the CRF, and severe cases will be reported to the ethical committee within 30 days.

A list of subject identification codes will be prepared to link the participants’ records to the records in the study database. The subject identification codes will also be used at the participants’ enrollment and on their CRFs. Limited information, such as sex and date of birth, may be used to identify the subjects or verify the list of subject identification codes, in accordance with all applicable laws and regulations. Finally, the participants’ data will be de-identified by the principal investigator or sub-investigators, and the data management will subsequently rely on the subject identification codes, which will not be shared with any outside parties. All study-related publications will be created in a format that prevents the participants from being personally identified.

### Analysis methods

#### Analysis population

The analysis dataset will include data from all enrolled participants who completed the staging laparoscopy, but without data from patients who withdrew their informed consent or did not complete the NIR-ICG imaging for any reason.

#### *Statistical analysi*s

The subjects’ baseline characteristics will be reported using summary statistics and distributions. Nominal variables will be reported as number and proportion. Continuous variables will be reported as the number of subjects, mean and standard deviation values, as well as minimum, median, and maximum values. The primary outcome analysis will evaluate the proportion and 95% confidence interval for the identification of at least one additional metastatic tumor using NIR-ICG imaging. The hypothesis testing will be based on a single sample, a null hypothesis of 0% efficacy, and a one-sided significance level of 2.5%. Efficacy is defined as the identification of at least one metastatic tumor that was not detected using standard white-light imaging. “Metastatic tumor” is defined as a histologically diagnosed lesion with adenocarcinoma. The sensitivity and specificity of white-light imaging and NIR-ICG imaging will be investigated. Safety outcomes will be evaluated using the proportion and two-sided 95% confidence interval based on a binomial distribution.

### Data monitoring and auditing

The principal investigator will appoint the relevant study monitors, who will perform verification based on direct access to the source documents, informed consent forms, medical charts, and CRFs. For non-participant data, the monitors will perform study monitoring before, during, and after the end of the study, and the details are described in the “Written procedure for implementation of study monitoring”. No auditing is planned as part of this exploratory study.

### Consent

The principal investigator or sub-investigators will prepare the informed consent form and other documents that will be used to obtain written informed consent. These documents will provide the potential subjects with opportunities to ask questions and ample time to decide whether or not to provide consent. Each participant will be required to confirm that they sufficiently understand the study’s purpose and protocol before providing voluntary consent to participate.

### Patient and public involvement

The patients and the public will not be involved in the study design, the recruitment procedure, or the study’s execution.

## Discussion

Laparotomy with curative intent for R-PDAC can occasionally identify small metastases in radiographically non-metastatic PDAC, as well as early metastasis during radiation therapy for LA-PDAC. However, laparotomy without pancreatic resection involves a needless large incision in R-PDAC patients, and postoperative complications may delay the start of chemotherapy. For example, we have reported that 25% of R-PDAC patients developed cancer recurrence within 6 months after curative resection, and this early recurrence was associated with a poor prognosis (median survival time: 8.8 month) [8]. This suggests that these patients might have had occult metastasis at the time of the laparotomy, which would then be considered needless major surgery.

Patients with LA-PDAC may also develop early distant metastasis after radiotherapy. For example, Chang et al.’s multicenter randomized study revealed metastatic disease-free survival of 6.1 months for LA-PDAC patients who underwent induction chemotherapy followed by chemoradiotherapy, with 25–30% of the enrolled patients experiencing disease progression with distant metastasis at 12 months after their registration (Chang JS, Mukherjee). Thus, radiographically occult metastasis may also lead to inappropriate radiotherapy for radiographically LA-PDAC patients who actually have advanced systemic disease.

Staging laparoscopy can help identify radiographically occult metastasis before treatment for PDAC. Furthermore, 29–58% of LA-PDAC patients have metastatic disease that was identified using staging laparoscopy, which prompted Satoi et al. to recommend staging laparoscopy as a routine examination for LA-PDAC patients [9]. Allen et al. have also reported that staging laparoscopy is a useful diagnostic method based on their meta-analysis, as it identified previously unidentified unresectable disease in 22% of R-PDAC patients [1]. Nevertheless, despite the potential utility of staging laparoscopy, they also reported that 18% of R-PDAC patients were found to have unresectable disease during a subsequent laparotomy. Therefore, greater diagnostic accuracy is needed for more effective pretreatment staging of PDAC.

Imaging using NRI-ICG can contribute to R0 resection of hepatocellular carcinoma and colorectal metastasis to the liver, with a high detection rate for hepatic lesions [10, 11]. Peritoneal metastasis can also be identified using NIR-ICG imaging [4], and several other studies have indicated that NIR-ICG imaging is useful in patients with PDAC or periampullary malignancies. For example, Handgraaf et al. reported that NIR-ICG imaging provided additional value based on its high negative predictive value in cases of pancreatic cancer [12], while Yokogawa et al. and Katada et al. reported detection rates of 16 and 15% for using NIR-ICG imaging to detect unsuspected metastasis from pancreatic cancer [13, 14]. Nevertheless, there are scarce data regarding the role of NIR-ICG imaging for staging PDAC. Therefore, the present study aims to establish the clinical utility of NIR-ICG imaging for identifying occult metastases in radiographically non-metastatic PDAC. If the efficacy of this approach is confirmed, this modality may help physicians and patients avoid needless major surgery or radiotherapy.

## Data Availability

Not applicable. This is a study protocol with no data.

## Abbreviations

CECT: Contrast-enhanced computed tomography
EOB-MRI: gadolinium ethoxybenzyl diethylenetriamine pentaacetic acid-enhanced magnetic resonance imaging
FDG-PET: 18F-flurodeoxyglucose positron emission tomography
ICG: Indocyanine green
NIR: Near infrared
PDAC: Pancreatic ductal adenocarcinoma
PWC: Peritoneal washing cytology
SL: Staging laparoscopy
